# Nature's Chemists: The Discovery and Engineering of Phytochemical Biosynthesis

**DOI:** 10.3389/fchem.2020.596479

**Published:** 2020-11-09

**Authors:** Kaouthar Eljounaidi, Benjamin R. Lichman

**Affiliations:** Centre for Novel Agricultural Products, Department of Biology, University of York, York, United Kingdom

**Keywords:** phytochemicals, gene discovery, biosynthetic pathway, bioactive chemicals, synthetic biology, metabolic engineering, natural products, biosynthesis

## Abstract

Plants produce a diverse array of natural products, many of which have high pharmaceutical value or therapeutic potential. However, these compounds often occur at low concentrations in uncultivated species. Producing phytochemicals in heterologous systems has the potential to address the bioavailability issues related to obtaining these molecules from their natural source. Plants are suitable heterologous systems for the production of valuable phytochemicals as they are autotrophic, derive energy and carbon from photosynthesis, and have similar cellular context to native producer plants. In this review we highlight the methods that are used to elucidate natural product biosynthetic pathways, including the approaches leading to proposing the sequence of enzymatic steps, selecting enzyme candidates and characterizing gene function. We will also discuss the advantages of using plant chasses as production platforms for high value phytochemicals. In addition, through this report we will assess the emerging metabolic engineering strategies that have been developed to enhance and optimize the production of natural and novel bioactive phytochemicals in heterologous plant systems.

## Introduction

Plants have a remarkable capacity to produce a wide array of specialized metabolites (also referred to as secondary metabolites or natural products), to support their defense and ecological adaptation. These phytochemicals exhibit a variety of bioactivities and many of them are of considerable pharmaceutical importance. Based on their biosynthetic origins, plant secondary metabolites can be divided into three major groups: terpenoids, alkaloids, and phenolics.

**Figure 1:**
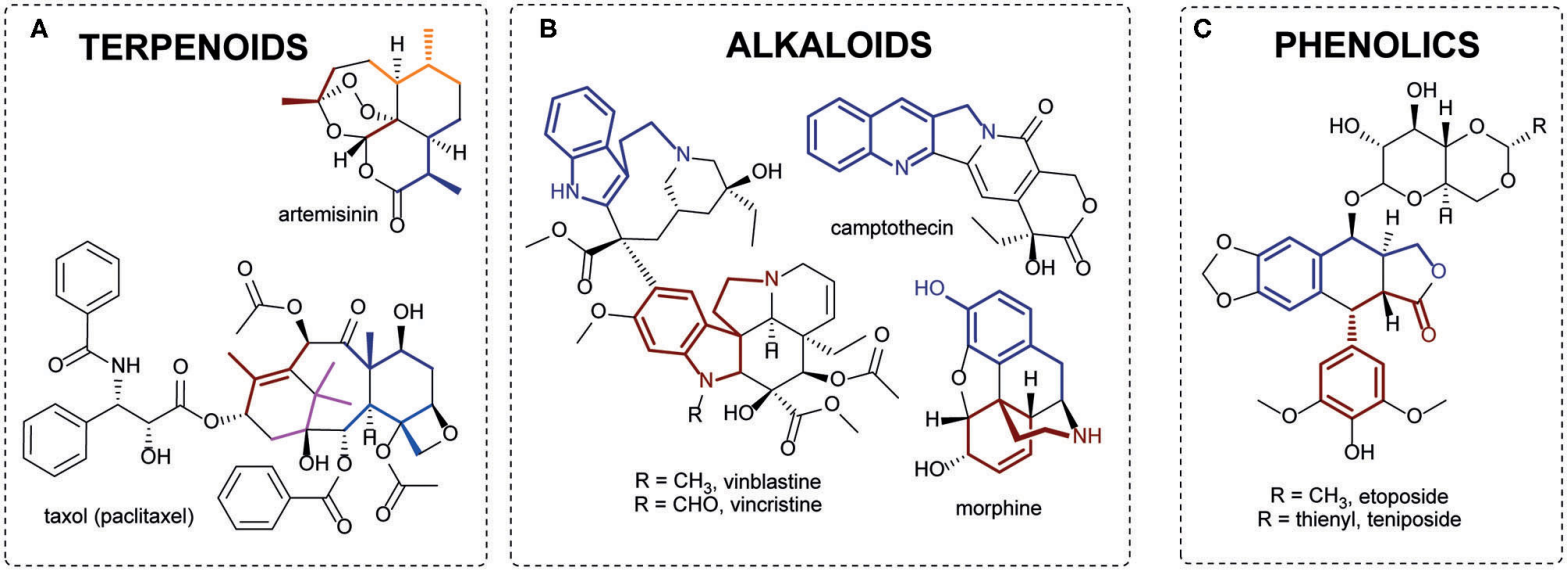
Examples of plant specialized metabolites of pharmaceutical significance. **(A)** Terpenoids: artemisinin and taxol. Isoprene units are highlighted with colors. **(B)** Alkaloids: camptothecin, vinblastine, and vincristine with tryptophan precursor highlighted; morphine tyrosine precursors highlighted. **(C)** Phenolics: etoposide and teniposide with phenylpropanoid units highlighted.

Terpenoids or isoprenoids are a structurally diverse class of plant specialized metabolites. They are derived by the repetitive fusion of branched five-carbon isopentanes, usually referred to as isoprene units. Many terpenoids have biological activities and are utilized as valuable pharmaceuticals. The most renowned terpenoid-based drugs include the antimalarial medicine artemisinin (*Artemisia annua*) and the anticancer drug Taxol (*Taxus brevifolia*) (Figure 1A). Another major class of secondary metabolites are alkaloids, nitrogen containing low-molecular-weight compounds, typically derived from amino acids (Lichman, 2020). Numerous alkaloids (or their derivatives) are employed as high value drugs, such as vincristine and vinblastine (anticancer) from Madagascar periwinkle (*Catharanthus roseus*), camptothecin (anticancer) from *Camptotheca acuminata* and morphine (analgesic drug) from opium poppy (Figure 1B). Phenolics are a large and diverse group of aromatic compounds which include the tannins, phenylpropanoid, anthocyanin and lignan subgroups. Etoposide and teniposide are among the most pharmaceutically important polyphenols (Figure 1C). These compounds are derivatives of podophyllotoxin, a lignan from mayapple (*Podophyllum peltatum*), and are widely used in chemotherapies. Alongside alkaloids, terpenoids, and phenolics, plants also produce other types of secondary metabolites, such as polyketides, cyanogenic glycosides and glucosinolates, of which many have pharmaceutical relevance.

Sourcing bioactive natural products from plants has many challenges. These metabolites are often found in low abundance or are produced in rare or slow-growing plant species, which limits the availability and accessibility of the active pharmaceutical agent and impacts market prices. In many cases, these molecules are very complex and are recalcitrant to chemical synthesis, as they contain many chiral centers or polycyclic rings (Nicolaou and Rigol, 2020). Even when synthesis is possible, it is not sustainable as it often relies on petrochemical feedstocks and/or environmentally unfriendly production processes (Lechner et al., 2016). This makes finding alternative sources for the supply of these valuable molecules necessary.

Increased access to phytochemicals can be achieved through a number of approaches. For instance, the biosynthetic capabilities of the natural producer may be enhanced through selective breeding programmes (Townsend et al., 2013) or through genetic modification and the creation of transgenic lines (Shen et al., 2018). Cell cultures may also enable greater control over the production of the compound of interest. For example, cambial meristematic cell cultures derived from *Taxus cuspidata* have been shown to produce high yields of the anticancer drug, paclitaxel (Taxol), while circumventing the obstacles routinely associated with the commercial growth of dedifferentiated plant cells (Lee et al., 2010).

Another alternative and increasingly popular approach is the transfer of biosynthetic pathways into heterologous systems such as plants (e.g., *Nicotiana benthamiana*) or microbes (e.g., *Saccharomyces cerevisiae* and *Escherichia coli*). The advantages of using microbes as production platforms for valuable chemicals include fast growth cycles, ease of genetic modification, and the fact that they offer a simplified product purification pipeline. The synthetic biology approaches used to produce plant metabolites in microbial organisms have been recently reviewed (Moses et al., 2017; Li et al., 2018). Plants, on the other hand, are photoautotrophs and do not require exogenous carbon sources, unlike typical microbial platforms. Plants also provide a cellular context similar to the native producer, which make them an attractive platform for the production of valuable chemicals.

In order to engineer valuable phytochemicals in microbe or plants, the first step is to uncover the biosynthetic pathway of the metabolite of interest. In this review we highlight the recent methods that have been used to elucidate natural product biosynthetic pathways, including the approaches leading to proposing the sequence of enzymatic steps, assigning enzyme family, as well as, gene function elucidation. We will also discuss the advantages of using plant chassis as a production platform for high value phytochemicals. In addition, through this report we will assess the emerging metabolic engineering strategies that have been developed to enhance and optimize the production of natural and novel bioactive phytochemicals in heterologous plant systems.

## Discovery of Specialized Metabolic Pathways

### Proposing a Biosynthetic Pathway

In order to identify the enzymes that underlie the biosynthesis of valuable plant natural products, it is first necessary to hypothesize a plausible sequence of enzyme catalyzed reactions that can lead from primary metabolism to the molecule of interest. Generally, biomimetic syntheses tend to use mild conditions and harness cascade-like reactions commonly found in biosynthetic pathways, hence providing clues for pathway elucidation (Yoder and Johnston, 2005).

If the metabolic pathway is incompletely described, isotope-labeling studies can be used to identify the unknown steps. Primary metabolites, such as amino acids, sugars, or even dioxygen and carbon dioxide, labeled with stable isotopes (e.g. ^2^H, ^13^C, ^15^N, and ^18^O), can be fed to the plant. The target specialized metabolite can then be assessed for isotope labeling by mass spectrometry (MS) or nuclear magnetic resonance (NMR) (Freund and Hegeman, 2017). This allows us to establish the identity of the starting material and identify its mode of incorporation. Strategically positioned isotopic labels can be used to probe the transformations and structural rearrangements that occur along the biosynthetic pathway.

For example, the incompletely described biosynthesis of camptothecin, a monoterpene indole alkaloid (MIA) from *Camptotheca acuminata*, was investigated using metabolite profiling and isotope labeling studies. The approach led to the identification of plausible intermediates for missing pathway steps and demonstrated that nearly all camptothecin pathway intermediates were present as multiple isomers (Sadre et al., 2016). Therefore, these metabolite focused strategies can lead to a hypothetical biosynthetic pathway made up of a set of chemically reasonable enzymatic conversions.

The next step is to assign possible enzyme classes to each hypothesized reaction based on characterized enzyme activities. For instance, a hydroxylation step will probably involve a cytochrome P450 enzyme or a 2-oxoglutarate dependent oxygenase (Mitchell and Weng, 2019), whilst a transfer of an amine group onto a carbonyl is likely to involve a PLP-dependent enzyme (Lee and Facchini, 2011). Once the hypothetical pathway has been populated with proposed enzyme classes, the gene discovery effort can begin.

### Integrative Approaches for Gene Identification

**Figure 2:**
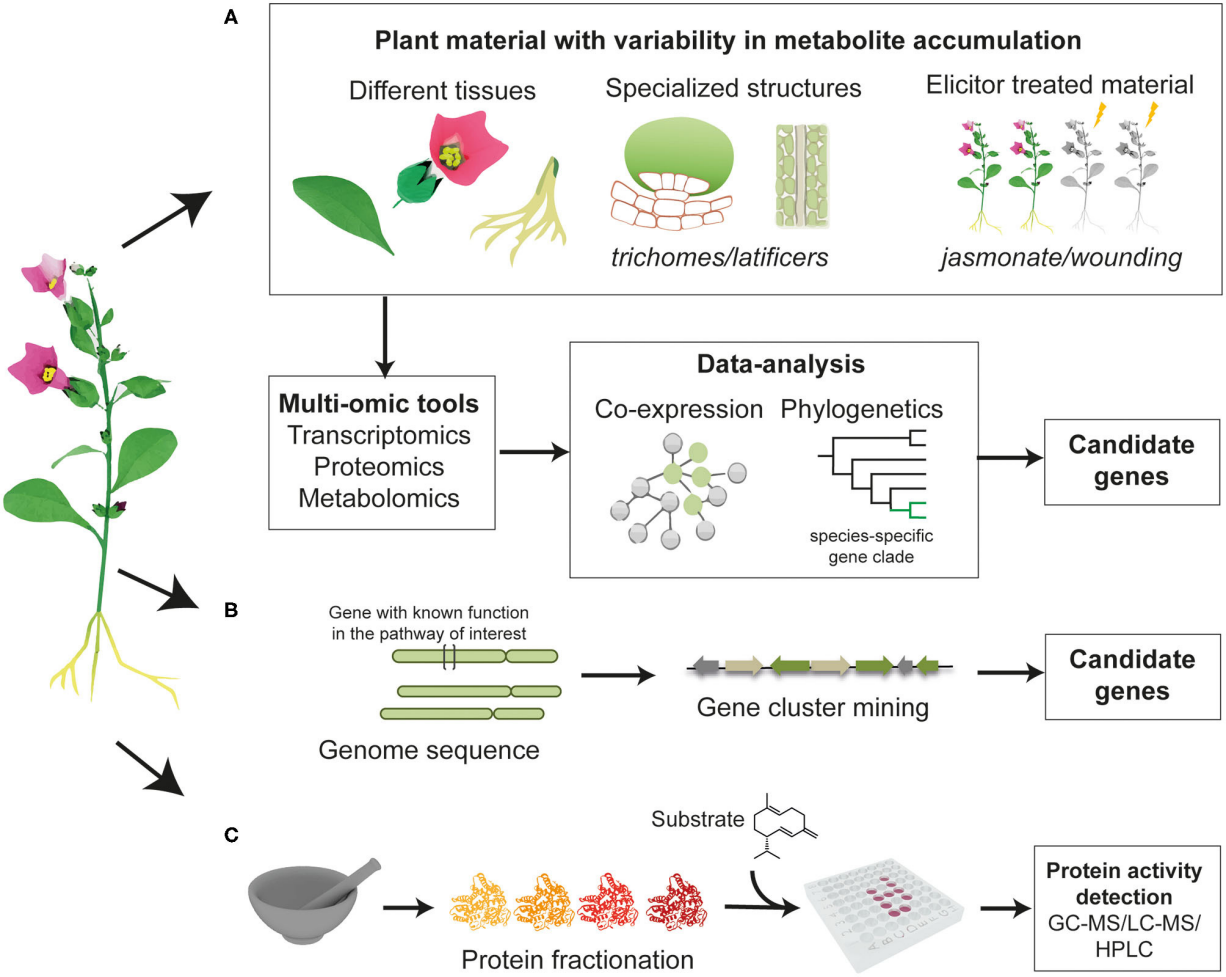
Overview of the approaches that can be employed to discover unknown enzymes in a biosynthetic pathway of interest. **(A)** Identification of candidate genes in plant material with difference in metabolites accumulation, through co-expression analysis or phylogenetic analysis to examine whether a gene clade is specific to the native plant producer. **(B)** Mining genomic data to look for genes that are physically localized in the vicinity of previously characterized enzymes (gene clusters). **(C)** Total protein purification and fractionation from the native plant material, followed by functional assays and proteomic identification of the active fraction.

The discovery of genes involved in the biosynthesis of phytochemicals typically requires the use of multi-omics technologies such as genomics, transcriptomics, proteomics, and metabolomics. Analysis of these data enable the identification of candidate genes that encode enzymes that catalyze biosynthetic steps (Figure 2).

The biosynthesis of natural products is regulated both during development and in response to various environmental stimuli. Transcriptomics can pinpoint differentially expressed genes across different types of tissues (or cells), different developmental stages or in elicitor-treated material. Candidate genes involved in biosynthesis can be identified through correlation of expression with metabolite accumulation, or through identification of co-expressed clusters in which genes involved in the same pathway share expression patterns across tissues (Figure 2A).

The hormone methyl jasmonate (MeJA) is known to elicit various species-specific specialized metabolic pathways. This feature has often been used to identify genes involved in the biosynthesis of various natural products. Cytochrome P450 (CYP728B70) catalyzing the oxidation step in triptolide biosynthesis, an abietane-type diterpenoid from *Tripterygium wilfordii*, was identified through an integrative gene prioritization approach (Tu et al., 2020). The prioritized candidate genes were highly expressed in MeJA-induced cells and/or in the root bark (main accumulation site of the diterpene), as well as, exhibited similar expression patterns to already characterized enzymes in the pathway; copalyl diphosphate synthase and miltiradiene synthase (Tu et al., 2020).

Various valuable plant natural products have been shown to accumulate in specific specialized structures, such as artemisinin in the glandular trichomes of *Artemisia annua* (Duke et al., 1994) and morphine in the laticifers of the aerial organs of opium poppy (*Papaver somniferum*) (Facchini and De Luca, 1995). Generating multi-omic data from these structures allows the identification of new biosynthetic enzymes involved in pathways of interest. For example, comparative proteomic analysis of trichomes and trichome-depleted leaves in catmint (*Nepeta mussinii*) led to the discovery of the unusual nepetalactol-related short-chain dehydrogenase enzymes (NEPS), which are enriched in the trichome and involved in the biosynthesis of volatile nepetalactones (Lichman et al., 2019).

Phylogenetic analysis can provide valuable information to further guide the candidate gene prioritization process. Genes involved in a biosynthetic pathway unique to a particular species are likely to be phylogenetically distinct. Therefore, building phylogenetic trees containing candidate genes and homologous genes from other species may reveal subclades unique to the producer plant. Key oxidases in limonoid biosynthesis in *Melia azedarach*; CYP71BQ5 and CYP71CD2 were identified through this strategy. These cytochrome P450 enzymes were found in a distinct subclade within the phylogenetic tree, comprising only *Melia azedarach* sequences, suggesting their involvement in this lineage-specific pathway (Hodgson et al., 2019).

Candidate gene selection may also be supplemented by mining whole genomes or partial genome sequences for genes physically localized in the vicinity of previously characterized enzymes (Figure 2B). It has been reported that genes involved in biosynthetic pathways of a number of natural products are organized in functional clusters within plant genomes (Roselli et al., 2017; Lichman et al., 2020; Liu et al., 2020). Features of plant biosynthetic gene clusters, how they form and how they are regulated have been recently reviewed (Nützmann et al., 2016, 2018). Another approach is total protein purification and fractionation, from the native plant material, followed by functional assays (Figure 2C). Protein fractions demonstrating the desired enzyme activity can be analyzed through protein-mass spectrometry and the identity of the enzyme determined by comparison with a predicted peptide database. The formation of thebaine, the first opiate alkaloid in the biosynthesis of codeine and morphine in opium poppy, can occur spontaneously from (7*S*)-salutaridinol 7-O-acetate. However, functional assays with total soluble protein isolated from opium poppy latex resulted in a 10-fold increase in the formation of thebaine. After protein fractionation and subjecting the active protein fraction to LC-MS/MS proteomics analysis, candidate genes were identified and were then tested through enzymatic assays, leading to the functional characterization of thebaine synthase (Chen et al., 2018).

Most of the aforementioned strategies, such as co-expression analysis, phylogenetics and genome mining, tend to be employed in an integrative way to allow efficient prioritization of candidate genes responsible for metabolic traits of interest. The next step is to verify the identity of the gene through functional assays.

### Functional Characterization of Candidate Genes

Two types of experiments are typically required to ascribe a gene to a biosynthetic pathway: enzyme activity assays and reverse genetics gene function validation. The activity of the encoded enzyme must be characterized; this is typically achieved using recombinant expression. However, activity alone is not sufficient to determine *in planta* function. Here, reverse genetics approaches such as gene silencing are necessary to verify the role of the gene in a pathway.

#### Pathway Elucidation in Microbial Platforms

**Figure 3:**
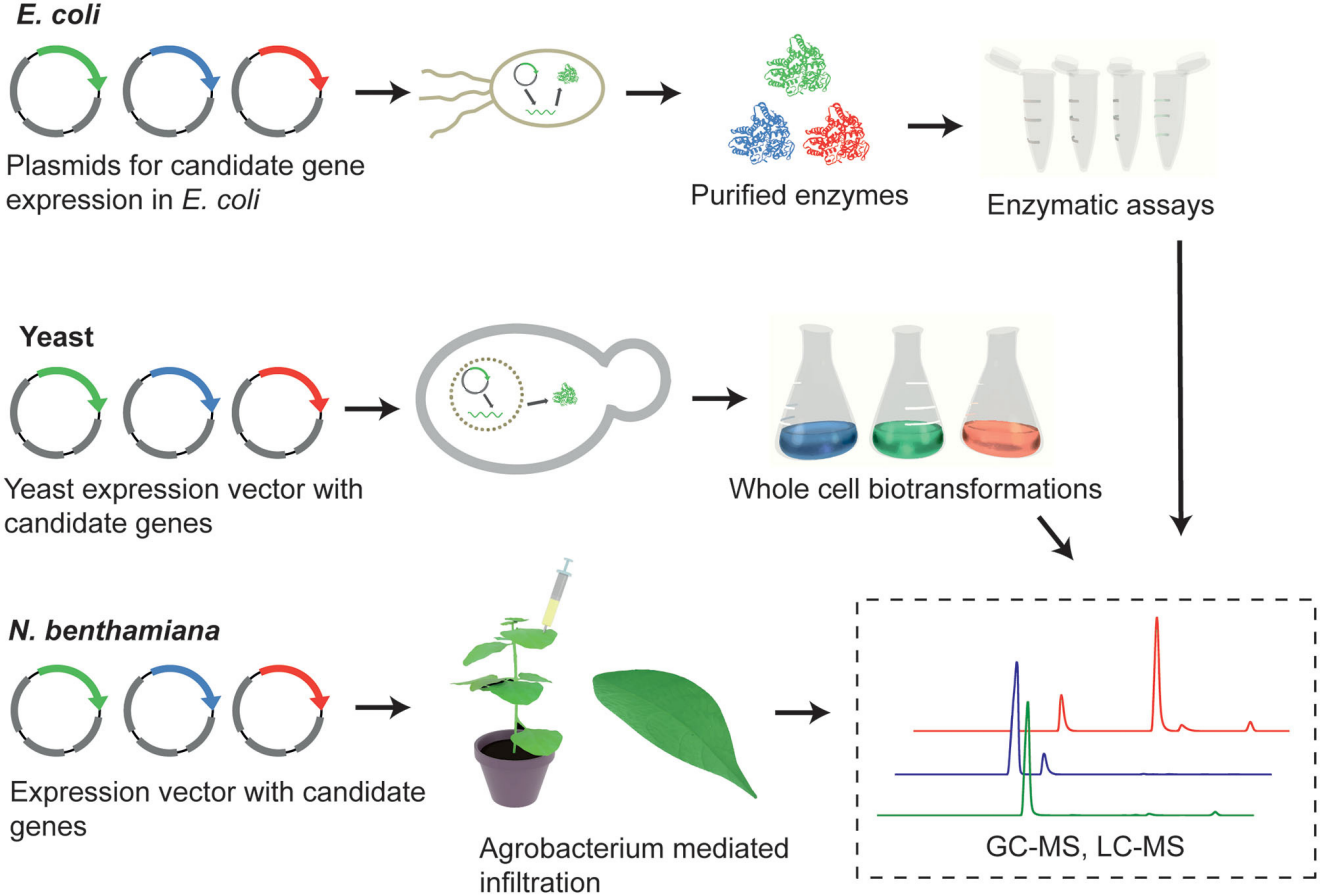
Overview of the functional characterization methods that can be utilized to determine gene function; enzymatic assays, heterologous expression of candidate biosynthetic genes in microbial host (yeast cells), and heterologous expression *in planta* (*Nicotiana benthamiana*).

Recombinant protein expression in *Escherichia coli* has been extensively used for the enzymatic characterization of biosynthetic genes. The advantages of employing this prokaryotic organism include fast growth kinetics and well-established molecular tools. Protein expression in *E. coli* is often followed by enzyme purification and *in vitro* assays. This enables the assessment of the biochemical activities of the candidate enzymes outside the complex cellular context (Caputi et al., 2018; Torrens-Spence et al., 2018; Lichman et al., 2019; Kim et al., 2020) (Figure 3).

However, several enzyme families frequently involved in specialized metabolism in plants are membrane-associated, such as cytochrome P450s and membrane-bound prenyltransferases, or require specific eukaryotic-type post-translational modifications. For expression of these proteins, *Saccharomyces cerevisiae* is preferred over *E. coli* because of its eukaryotic cell architecture, including the availability of more suitable protein post-translational mechanisms. Microsomal fractions of yeast cells containing active recombinant proteins can be prepared and utilized for subsequent biochemical assays (Levsh et al., 2019).

Alternatively, it is possible to exploit endogenous yeast metabolism and examine the enzymatic activity in an *in vivo* context, without purification. This method relies on the available precursors and substrates within native yeast pathways (Figure 3). This approach may be preferred over *in vitro* assays if enzyme purification is challenging or higher throughput is required. If the substrate of interest is not available in yeast, an exogenously supplied substrate can be used to build the desired secondary metabolite (Tu et al., 2020). Another option is to engineer yeast strains through genome engineering (e.g., CRISPR or homologous recombination) to accumulate specific precursors that are not naturally present in yeast (Luo et al., 2019; Munakata et al., 2019), or to shutdown endogenous competitive branches that deplete the precursor of interest (Moses et al., 2014).

#### Pathway Elucidation in *Nicotiana benthamiana*

*Nicotiana benthamiana* is a practical heterologous expression system for plant natural product biosynthetic pathways (Figure 3). It is highly amenable to *Agrobacterium*-mediated transformation, allowing the transient expression of one or, simultaneously, multiple gene(s) of interest. This plant also provides a cellular context that is similar to the native producer plant. This characteristic allows the expressed proteins to be properly folded and targeted to the correct subcellular compartment, as well as provides a natural supply of chemical precursors; coenzymes, and cofactors.

Heterologous expression of candidate genes in *N. benthama* has led to the discovery of a variety of enzymes underlying the biosynthesis of diverse high value plant-derived compounds (Levsh et al., 2019; Pluskal et al., 2019). The plant can also be used to reconstitute whole pathways for preparation of high-value compounds. For example, eight genes from *Gloriosa superba* involved in the biosynthesis of the alkaloid colchicine were discovered using *N. benthamiana* as a screening tool. Subsequently, the newly discovered genes plus eight previously described genes were used to engineer *N. benthamiana* to synthesise *N*-formyldemecolcine, a colchicine precursor, from phenylalanine and tyrosine (Nett et al., 2020). The use of *N. benthamiana* for the heterologous production of whole pathways is discussed further in section Plants as Chasses for Production of High-Value Phytochemicals.

#### Virus Induced Gene Silencing

Virus-induced gene silencing (VIGS) is a reverse genetics approach commonly employed for the *in vivo* functional characterization of candidate biosynthetic genes (Moglia et al., 2016). VIGS provides direct evidence of the biological role of the gene through a loss-of-function mechanism. The system takes advantage of the plant's homology-dependent defense mechanisms in response to attack by viruses (Hileman et al., 2005). Inoculating the plant with a construct containing viral RNA and a fragment of the target gene will trigger the degradation or the inhibition of translation of the correspondent RNA, leading to the silencing of the gene of interest. The knock-down of specific genes through this system will reveal their involvement in the biosynthesis of the target natural product. The system was established and used in a variety of plants such as Madagascan periwinkle (*Catharanthus roseus*) (Qu et al., 2019) and opium poppy (Chen et al., 2018). However, a species-specific set up of the method is needed for each newly studied plant by, for instance, identifying suitable virus for the plant and efficient inoculation methods (Courdavault et al., 2020).

## Plants as Chasses for the Production of High-Value Phytochemicals

Expressing genes in heterologous plant systems not only enables pathway elucidation and gene function determination, but also provides new opportunities to increase and diversify the production of high-value bioactive phytochemicals. As mentioned above, *N. benthamiana* represents a valuable tool for the heterologous production of phytochemicals. For example, 16 biosynthetic genes from Himalayan mayapple (*Podophyllum hexandrum*) were transferred to *N. benthamiana* to produce precursors of the chemotherapeutic drug etoposide at milligram-scale (Schultz et al., 2019).

A major advantage of using *N. benthamina* as a production platform is the rapidity of the process; no transgenic plants need to be generated. Turnaround times are comparable to microbial systems, with the detection of products possible a few days after agroinfiltration. The process can be scaled up using vacuum agroinfiltration to reach gram-scale production of phytochemicals (Reed et al., 2017).

### Optimizing Plant-Based Production of Phytochemicals

Diverse strategies have been used to enhance and optimize the production of phytochemicals *in planta*. Among these strategies include overexpressing yield-boosting enzymes such as tHMGR (truncated 3-hydroxy-3-methylglutaryl-CoA reductase, key rate-limiting enzyme in mevalonate pathway) (Reed et al., 2017) or 1-deoxy-d-xylulose 5-phosphate synthase (DXS), the first committed MEP pathway enzyme (Brückner and Tissier, 2013). Another method that can potentially lead to increased production is the suppression of competitive pathways through inactivation of endogenous genes by virus-induced gene silencing (Hasan et al., 2014) or RNA interference (Cankar et al., 2015).

The biosynthesis of natural products in plants is highly compartmentalized with different steps of biosynthetic pathways occurring in different subcellular localization (Heinig et al., 2013). To enhance the production of phytochemicals of interest, an alternative method is to alter the subcellular location of heterologously expressed enzymes by addition, removal or modification of target peptides (Reed and Osbourn, 2018). Various studies have shown the potential of this strategy in enhancing the production, with targeting biosynthetic enzymes in different subcellular compartments. Dong et al. (2016) reported that the targeting of geraniol synthase (GES) from *Valeriana officinalis* in the chloroplasts of *N. benthamina* increased the production of GES products compared to the mitochondrial- and cytosolic-targeted GES.

### Emerging Metabolic Engineering Strategies

The ability of plant specialized structures, such as glandular trichomes, to synthesize and store hydrophobic and toxic metabolites has the potential to make engineering the production of these metabolites in plants more advantageous (Huchelmann et al., 2017). Conditions in trichomes and other structures may even be vital for the formation of the target compound: the final oxidative steps in the biosynthesis of artemisinin are non-enzymatic and require the non-aqueous environment of the subapical cavity of glandular trichomes to proceed (Czechowski et al., 2016). These findings highlight the challenges of producing specific secondary metabolites in microbial platforms that lack this level of structural complexity.

In addition, engineering metabolic pathways in plants is sometimes hindered by toxicity issues and growth defects, due to the cytotoxicity of the engineered compounds or the depleted pools of precursors, necessary for central metabolism. Expressing these pathways in a cell-type specific way, by taking advantage of the separation of trichomes from the rest of the plant, might offer a solution for these issues (Tissier et al., 2012). Efforts to produce secondary metabolites in trichomes through the use of trichome-specific promoters have been reported, such as; taxadiene in *N. sylvestris*, and casbene (diterpene from *Ricinus communis*), in *N. tabacum* (Table 1) (Rontein et al., 2008; Tissier et al., 2012).

**Table 1:** Examples of engineered cellular/subcellular localization of phytochemical production in *Nicotiana* sp.

**Host plant**	**Localization**	**Engineering strategy**	**Compound class**	**Compound accumulation**	**References**
*N. benthamiana*	Synthetic hydrophobic organelles	Transient expression; fusing terpenoid enzymes to a microalgal lipid droplet surface protein	Diterpenoids	2.5 fold increase of target diterpenoids/diterpenoids acids	Sadre et al., 2019
*N. benthamiana*	Lipid bodies	Transient expression; Co-expression of α-bisabolol synthase with fatty acids biosynthesis regulators	Sesquiterpenoids	2–4 fold increase of; α-bisabolol, (E)-β -caryophyllene and α-barbatene.	Delatte et al., 2018
*N. tabacum*	Plastids	Stable transformation; plastome and nuclear transformation with artemisinic acid biosynthetic genes and other enzymes known to affect the metabolic flux	Sesquiterpenoids (artemisinic acid)	Accumulation of more than 120 mg/kg FW of Artemisinic acid.	Fuentes et al., 2016
*N. tabacum*	Glandular trichomes	Stable transformation; Casbene synthase/Trichome-specific promoters	Diterpenoids (casbene)	Accumulation of 1 mg/g FW of casbene	Tissier et al., 2012
*N. sylvestris*	Glandular trichomes	Stable transformation; taxadiene synthase/Trichome-specific promoters	Diterpenoids (Taxadiene)	Accumulation of 100 μg/g FW of taxadiene	Tissier et al., 2012

Engineering plastids in order to optimize the yields of valuable phytochemicals has been explored. Plastid engineering has various advantages over nuclear genome engineering including an efficient homologous recombination machinery, as well as the potential for greater gene expression levels (Bock, 2015; Boehm and Bock, 2019). To enhance the yield of artemisinic acid production (the precursor for the anti-malaria drug: artemisinin), a synthetic biology strategy has been developed combining plastome and nuclear transformation (Fuentes et al., 2016). The artemisinic acid metabolic pathway was introduced into the chloroplast genome of *N. tabacum* plants. Subsequently, enzymes known to affect the metabolic flux were introduced through nuclear transformation (Table 1). This strategy led to identifying plants that produce high amounts of artemisinic acid (more than 120 mg of per kg biomass) (Fuentes et al., 2016; Jensen and Scharff, 2019).

A newly emerging metabolic engineering strategy is engineering synthetic hydrophobic droplets within cells that enable the accumulation and storage of lipophilic compounds such as terpenoids. For example, the synthesis of lipid droplets in transient *N. benthamiana* system was enhanced through the ectopic production of a regulator of plastid fatty acid biosynthesis and a microalgal lipid droplet surface protein. Biosynthetic steps were anchored onto the surface of the lipid droplets and high-value sesqui- or diterpenoids were efficiently produced. These engineered lipid droplets may potentially facilitate terpenoid extraction from the plant, through “trapping” of the target molecules in the oil fraction (Table 1) (Sadre et al., 2019). Another study demonstrated the potential of lipid bodies as hydrophobic storage organelle for sesquiterpenes molecules such as α-bisabolol (Delatte et al., 2018).

CRISPR and Cas9-associated protein systems are emerging as powerful tools to study gene function and to improve specific traits in plant species. Knocking out biosynthetic genes using CRISPR/Cas9 system has been reported in *Salvia miltiorrhiza* (tanshinone, diterpenoid) (Li et al., 2017) and *Papaver somniferum* L. (benzylisoquinoline alkaloids) (Alagoz et al., 2016). Setting up CRISPR/Cas9 systems in diverse plant species will be useful for gene function elucidation studies and will also guide future efforts to improve the yield of phytochemicals of interest in valuable medicinal plants. CRISPR/Cas9 systems also have the potential to shut down competing side branches of metabolic pathways within plants, leading to improved yields of the target compound.

### Combinatorial Biosynthesis

Engineering new-to-nature metabolic pathways in plants offers the possibility to expand the natural chemical diversity of plants and create a subset of novel chemical structures with new or improved bioactivities. Combinatorial biosynthesis is an engineering strategy, based on combining biosynthetic genes from different sources in a single host, thereby establishing new enzyme–substrate combinations, that enables the generation of a suite of related compounds including many that are new-to-nature (Arendt et al., 2016). This strategy takes advantage of the hypothesis that enzymes involved in plant natural product biosynthesis have inherent relaxed substrate specificity and are often able to transform non-native substrates. Different studies aiming to explore the potential of this approach in creating novel compounds have been reported (Table 2).

**Table 2:** Examples of novel compounds produced through combinatorial biosynthesis using transient expression in *N. benthamiana*.

**Source of enzymes**	**Enzymes**	**Compound class**	**Obtained compounds**	**References**
*Tanacetum parthenium/Artemisia annua*	Germacrene A synthase (GAS) Germacrene A oxidase (GAO) Costunolide synthase (COS) Parthenolide synthase (PTS) aldehyde Δ11(13) double bond reductase2 (DBR2)	Sesquiterpenoids	Costunolide and parthenolide derivatives	Kashkooli Beyraghdar et al., 2019
Oat, licorice, soy bean, barrel clover	β-amyrin synthase and β-amyrin-oxidizing P450s	Triterpenoids	β-amyrin derivatives	Reed et al., 2017
Various species	Class I/II diterpene synthases	Diterpenoids	Diverse diterpene skeletons	Andersen-Ranberg et al., 2016
*Setaria italica, Sorghum bicolor, Lotus japonicus, Brassica rapa*	Cytochrome P450s Glucosinolate biosynthetic genes	Isothiocyanate	Crucifalexins	Calgaro-Kozina et al., 2020

Co-agroinfiltration of *N. benthamiana* with different expression constructs is an efficient method to test numerous enzyme combinations (Reed and Osbourn, 2018). This approach was used to generate novel β-amyrin derivatives through the expression of combinations of β-amyrin synthase and β-amyrin-oxidizing P450s from various plant species (Reed et al., 2017). In another study, the stereoselective biosynthesis of over 50 diterpene skeletons including natural variants and novel compounds was achieved *via* transient expression of combinations of class I and II diterpene synthases in *N. benthamiana* (Andersen-Ranberg et al., 2016). These terpene synthases were isolated from seven different diterpenoids producing plant species.

The generation of costunolide and parthenolide derivatives was achieved by applying a combinatorial metabolic engineering approach expressing different enzymes involved in sesquiterpenoids biosynthesis from *Tanacetum parthenium* and *Artemisia annua* (Table 2) (Kashkooli Beyraghdar et al., 2019). In a recent study, diverse cytochrome P450s from *Setaria italica, Sorghum bicolor, Lotus japonicus*, as well as glucosinolate biosynthetic genes from *Brassica rapa*, were heterologously expressed in *N. benthamiana*. This expression generated new-to-nature compounds with potent antifungal activity, called crucifalexins. This report showed that by broadening the set of primary metabolites that can be utilized by the core biosynthetic pathway of brassinin (isothiocyanate), it is possible to synthesize novel molecules with enhanced proprieties (Calgaro-Kozina et al., 2020).

## Conclusion

Phytochemicals were foundational to the emergence of organic chemistry and the early development of pharmaceuticals. The structural complexity and bioactivity of phytochemicals will ensure their continued relevance in synthetic chemistry and drug discovery. With the maturity of genetic and biochemical tools for gene discovery, characterization and engineering, we are entering into a new synthetic era of phytochemistry, in which biosynthetic pathways are elucidated and high-value phytochemicals can be produced in synthetic biological chasses.

An integrative approach to gene prioritization, combining genomic transcriptomic and metabolomics, has become an efficient method for the identification of candidate biosynthetic genes. Characterization of genes through *in vitro* assays, coupled with *in vivo* validation through RNAi or VIGS, subsequently enables verification of candidate gene function. Once the pathway is uncovered, the heterologous *in planta* expression of the biosynthetic pathway is made possible, with many emerging engineering strategies available to enhance or diversify the production of the target compounds. These advances open the door to a future where diverse phytochemicals with new bioactivities and unusual structures can be routinely produced and purified from heterologous plant systems, aiding drug discovery efforts and leading to the scalable and sustainable production of valuable phytochemicals.

## Author Contributions

KE conceived of the manuscript and wrote the first draft. Both authors prepared figures, edited text, and approved the manuscript for submission.

## Conflict of Interest

The authors declare that the research was conducted in the absence of any commercial or financial relationships that could be construed as a potential conflict of interest.
